# Central venous pressure swing outperforms diaphragm ultrasound as a measure of inspiratory effort during pressure support ventilation in COVID-19 patients

**DOI:** 10.1007/s10877-021-00674-4

**Published:** 2021-02-26

**Authors:** Sergio Lassola, Sara Miori, Andrea Sanna, Alberto Cucino, Sandra Magnoni, Michele Umbrello

**Affiliations:** 1 SC Anestesia e Rianimazione 1, Ospedale Santa Chiara, Trento, Italy; 2 SC Anestesia e Rianimazione II, Ospedale San Carlo Borromeo, ASST Santi Paolo e Carlo, Milano, Italy

**Keywords:** Pressure support ventilation, Esophageal pressure, Diaphragm ultrasound, COVID-19, Weaning

## Abstract

**Purpose:**

The COVID-19-related shortage of ICU beds magnified the need of tools to properly titrate the ventilator assistance. We investigated whether bedside-available indices such as the ultrasonographic changes in diaphragm thickening ratio (TR) and the tidal swing in central venous pressure (ΔCVP) are reliable estimates of inspiratory effort, assessed as the tidal swing in esophageal pressure (ΔPes).

**Methods:**

Prospective, observational clinical investigation in the intensive care unit of a tertiary care Hospital. Fourteen critically-ill patients were enrolled (age 64 ± 7 years, BMI 29 ± 4 kg/m^2^), after 6 [3; 9] days from onset of assisted ventilation. A three-level pressure support trial was performed, at 10 (PS10), 5 (PS5) and 0 cmH_2_O (PS0). In each step, the esophageal and central venous pressure tidal swing were recorded, as well as diaphragm ultrasound.

**Results:**

The reduction of pressure support was associated with an increased respiratory rate and a reduced tidal volume, while minute ventilation was unchanged. ΔPes significantly increased with reducing support (5 [3; 8] vs. 8 [14; 13] vs. 12 [6; 16] cmH_2_O, p < 0.0001), as did the diaphragm TR (9.2 ± 6.1 vs. 17.6 ± 7.2 vs. 28.0 ± 10.0%, p < 0.0001) and the ΔCVP (4 [3; 7] vs. 8 [5; 9] vs. 10 [7; 11] cmH_2_O, p < 0.0001). ΔCVP was significantly associated with ΔPes (R^2^ = 0.810, p < 0.001), as was diaphragm TR, albeit with a lower coefficient of determination (R^2^ = 0.399, p < 0.001).

**Conclusions:**

In patients with COVID-19-associated respiratory failure undergoing assisted mechanical ventilation, ΔCVP is a better estimate of inspiratory effort than diaphragm ultrasound.

**Supplementary Information:**

The online version contains supplementary material available at 10.1007/s10877-021-00674-4.

## Introduction

In March 2020, the outbreak of Coronavirus disease 2019 (COVID-19)-related acute hypoxaemic respiratory failure widely stretched the healthcare systems worldwide beyond their capacities [1]. Several papers summarized the supportive care for the disease [2–5]; however, the vast majority is limited to the management of the acute phase, and weaning from mechanical ventilation is only marginally treated.

Weaning of mechanical ventilation has been defined as the process during which the work of breathing is progressively transferred from the ventilator back to the patient, as soon as the condition that caused respiratory failure has started to improve [6]. Pressure Support Ventilation is the assisted ventilation modality most widely used during the weaning phase [7, 8], in which the work of breathing ventilation is shared between the ventilator and the patient [9]. Since an excessive unloading of the diaphragm may lead to the development of disuse atrophy, while an insufficient ventilator assistance may be associated vigorous spontaneous efforts and the consequent co-development of under assistance diaphragm myotrauma and self-inflicted lung injury [10, 11], an acceptable level of muscle unloading while preserving spontaneous breathing is generally suggested as the most reasonable strategy to avoid complications and achieve a successful weaning from mechanical ventilation [12].

Nevertheless, an adequate assessment of patient inspiratory effort from physical examination or ventilator waveforms is often difficult [13] and direct measures are required to properly titrate the level of support. Esophageal pressure (Pes) represents the reference method for measuring the pressure generated by the respiratory muscles [14, 15]. We recently demonstrated how bedside assessment of the tidal swing in esophageal pressure (ΔPes) is an adequate estimate of inspiratory effort. However, such calculation requires an esophageal catheter, which is only seldom used in the everyday clinical practice [16], and is still generally regarded as a research tool; easier, bedside-available tools for the assessment of patient effort are required for everyday clinical practice, and especially more so in a situation of unbalance between the number of severe patients admitted and shortage of ICU beds and monitoring devices [17].

Since the superior vena cava is a highly compliant vein inside the thorax, the tidal swing of central venous pressure (ΔCVP) has long been known as a reasonable surrogate for the ΔPes in spontaneously breathing subjects and in patients undergoing assisted mechanical ventilation [18–20]. However, limited evidence is reported as to the use of ΔCVP to assess patient effort. Moreover, since transmission of the intrathoracic pressure to the superior vena cava may depend upon the filling state [21], we hypothesized that the relationship between the inspiratory effort and the ΔCVP could be affected by the value of CVP (both absolute and transmural).

Diaphragm ultrasound has recently been shown a potential tool for monitoring the respiratory effort of critically ill patients [22, 23]. However, the relationship between diaphragm thickening and respiratory effort is far from being completely understood, as it may be influenced by several factors, such as the thoracoabdominal pattern of breathing and the mechanical characteristics of the respiratory system, to the extent that the degree of thickening may vary strongly between patients at a given level of diaphragm effort [24].

The aim of the present study was to assess the comparative performance of diaphragm TR and ΔCVP to estimate inspiratory effort, as measured by the ΔPes, in a cohort of consecutive patients with COVID-19 acute respiratory failure undergoing pressure support ventilation during their weaning phase. Secondary outcome was the effect of absolute and transmural CVP on the relationship between ΔCVP and ΔPes, and the diagnostic ability of diaphragm TR and ΔCVP to identify a high or a low inspiratory effort, defined as by specific thresholds of ΔPes.

## Materials and methods

### Subjects

Consecutive patients were enrolled if they had been admitted for acute respiratory failure in COVID-19, and undergoing PSV with a positive end-expiratory pressure (PEEP) > 5 cmH_2_O. Exclusion criteria were: hemodynamic instability requiring vasopressors, hypoxemia requiring PEEP > 10 cmH_2_O and/or FiO_2_ > 60%, PS > 10 cmH_2_O, Richmond Agitation and Sedation Scale score <  − 1, history of COPD. Ethical approval for this study (Rep. Int. 11649/2020) was provided by the Comitato Etico per le Sperimentazioni Cliniche of the Azienda Provinciale per i Servizi Sanitari di Trento (Chairperson dott. Angelo del Favero) on 22 June 2020; written informed consent was obtained according to Italian regulations.

### Measurement

**Fig. 1:**
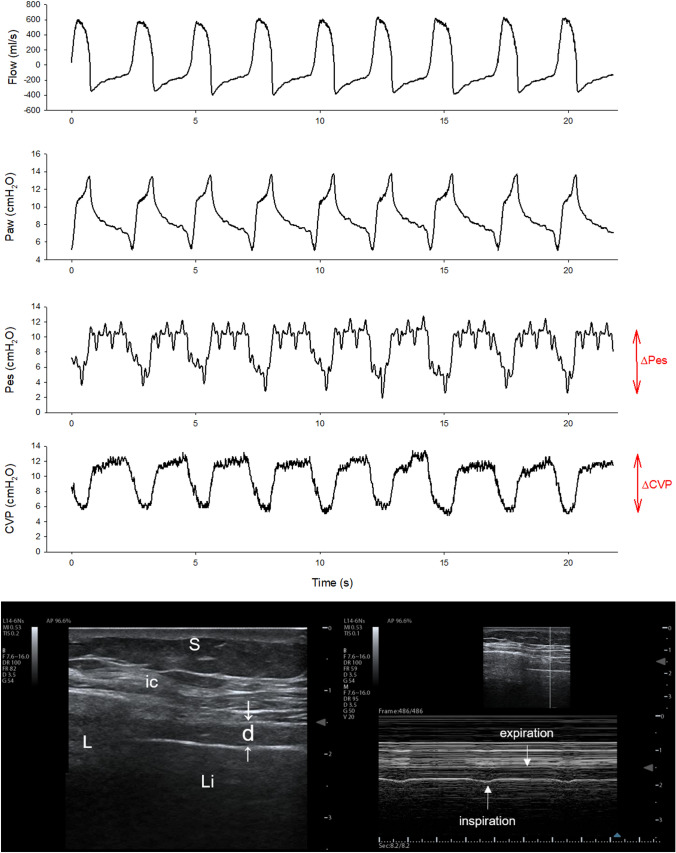
Inspiratory effort measurements in a representative patient. The upper part of the figure shows the flow, airway pressure (Paw), esophageal pressure (Pes) and central venous pressure (CVP) in a representative patient. The negativization of intrathoracic pressure during inspiration lowers the esophageal and central venous pressures (ΔPes and ΔCVP, respectively). The lower panel shows the ultrasonographic view of the diaphragm: on the left side a B-mode scan of the diaphragm in the zone of apposition is performed. The diaphragm (d) is identified as a three-layer structure (non-echogenic central layer bordered by two echogenic layers, the peritoneum and the diaphragmatic pleurae, indicated by the white arrows). *ic* intercostal muscles, *L* lung, *Li* liver, *S* subcutaneous tissue. On the right side, the M-mode image of diaphragm thickening during inspiration is shown, allowing for the calculation of the thickening ratio

Before enrolment in the study, all patients were treated according to National and local recommendations [25]. In particular, whenever a patient improved his/her oxygenation (PaO_2_/FiO_2_ > 200 with a PEEP ≤ 12 cmH_2_O in supine position for at least 12 h) interruption of muscle relaxant use was suggested, targeting a light level of sedation and attempting assisted ventilation. An initial pressure support level of 8–12 cmH_2_O was suggested, to obtain a VT between 5 and 8 ml/Kg predicted body weight; subsequent support titration was suggested to maintain respiratory rate < 35 breaths per minute. The assessment of P0.1 was advised to identify patients with a high respiratory drive. Figure 1 shows the measurement of inspiratory effort in a representative patient.

Patients were studied in the semirecumbent position. The lungs of the patients were ventilated in pressure support with the following settings: flow-triggering at 2 l/min, pressure ramp 200 ms, cycling-off at 25% of the peak inspiratory flow. Automatic-tube compensation was not used.

Esophageal pressure was measured using a balloon, graduated feeding catheter (NutriVent®, Mirandola, Modena, Italy), which was positioned under general anesthesia during mechanical ventilation. To check for the correct position of the esophageal balloon, a dynamic occlusion test was performed to ensure that Pes was changing in concert with the airway pressure (Paw) when making breathing against a closed airway [26]. The dynamic occlusion test was performed by a dedicated monitor (OptiVent®, Mirandola, Modena, Italy) which provides the level of confidence related to the quality of the occlusion maneuver and the R-square value of the relationship between Paw and Pes. The optimal inflation volume of the balloon was obtained by means of a specific calibration procedure carried out by the OptiVent® system, which inflates the balloon to different volumes while recording the respective pressure values.

CVP was measured from the distal port of a triple-lumen central venous catheter, whose distal end was located radiographically in the superior vena cava. The distal port was connected to a differential pressure transducer which was filled with a 0.9% saline solution. CVP measurements were zeroed at mid-thoracic position at the level of the fifth rib [27], and the value was taken during either end-inspiration or end-expiration, at the base of “c” wave [28]. Transmural CVP was calculated as the difference between CVP and Pes [29]. ΔCVP was recorded on a dedicated multi-parametric monitor (Carescape B850, GE Healthcare, Little Chalfont, UK).

### Pattern of breathing and respiratory effort

In each step, tidal volume, respiratory rate, minute ventilation, and the tidal swing in esophageal and central venous pressures (ΔPes and ΔCVP, respectively) were measured. Mean values were computed over three consecutive breaths. End-expiratory and end-inspiratory occlusions were performed to measure P0.1 [30] and Pmusc, index [31]: the airway pressure decrease in the first 100 ms after the onset of inspiration following an end-expiratory occlusion (P0.1) was measured, reflecting the patient’s respiratory drive. The estimated pressure developed by the inspiratory muscles at the end of an inspiratory effort (Pmusc), expressed as the Pmusc, index (PMI) was calculated as follows:

$${\text{PMI}} = {\text{ Pel}},{\text{rsi }}{-} \, \left( {{\text{PEEP}} + {\text{PS}}} \right)$$

where Pel, rsi is the elastic recoil pressure of the respiratory system at the end of inspiration, measured as the airway plateau pressure during an end-inspiratory occlusion maneuver.

### Ultrasonographic measurements

Ultrasonography was performed by the same trained operator (AS), with 5 years of experience and qualifications in respiratory ultrasound, using a General Electrics Vivid T8 Ultrasound System with a 12 MHz linear probe (GE Healthcare, Little Chalfont, UK). Images were recorded for a subsequent, computer-assisted quantitative analysis by a trained investigator (SM), unaware of the ventilatory condition.

Diaphragm thickness was assessed in the zone of apposition of the diaphragm to the rib cage. The linear probe was placed above the right 10^th^ rib in the mid-axillary line, as previously described [22]. The inferior border of the costophrenic sinus was identified as the transition from the artefactual representation of the lung to the visualization of the liver.

Three subsequent measures were averaged. The diaphragm thickening ratio (TR) was calculated as:

$$TR = \frac{{\left( {end{\text{ - }}inspiratory~\,thickness - end{\text{ - }}expiratory\,~thickness~} \right)}}{{end{\text{ - }}expiratory~\,thickness}}*100$$

### Protocol

Patients who were judged ready for a spontaneous breathing trial [32], underwent a trial of three levels of PSV, lasting 30 min each. The first level was set at 10 cmH_2_O (PS10). Pressure support was then reduced to 5 (PS5) and 0 cmH_2_O (PS0), in this order. PEEP and FiO_2_ were unchanged, as was the sedation level. During the last 5 min of each step, the pattern of breathing and indices of respiratory effort were assessed, diaphragm ultrasound was performed, and hemodynamic parameters were recorded. To avoid the confounding effect of the possible development of fatigue at low levels of assistance, which might have then influenced the following steps, we decided to perform a decremental pressure-support test and we did not randomize the sequence of pressure support levels. The protocol was allowed to be stopped, if patients developed signs of respiratory distress (respiratory rate > 35 breaths/min, SpO_2_ < 90%, heart rate > 140 beats/min, systolic blood pressure > 180 mmHg, diaphoresis or anxiety).

### Statistics

Since, to our knowledge, no previous publications have addressed a similar topic, a formal sample size calculation was not performed, and we enrolled a convenience sample of consecutive patients with a similar size to other physiological investigations. Data were analyzed using Stata 13.0 (StataCorp, College Station, Texas, USA) for Windows. Normality was assessed by the Shapiro-Francia test. Descriptive results are reported as mean (standard deviation) if normally distributed, or median [25–75th percentiles] otherwise. The analysis on the variables recorded over the three steps (PS10, PS5, PS0) was performed by analysis of variance for repeated measurements, with step as a within-subject factor, and the statistical significance of the within-subject factors was corrected with the Greenhouse–Geisser method. Non-parametric variables were analyzed using Friedman test. Pairwise, post-hoc multiple comparisons were carried out according to Tukey method. Regression was conducted by a linear, fixed-effects model for repeated measures to deal with the longitudinal structure of our data set (patients with repeated measurements over time). The association between variables was expressed as the coefficient of determination (R^2^). The diagnostic performance of the CVP swing and diaphragm TR for detecting either a low or a high inspiratory effort (arbitrarily defined as an esophageal pressure swing < 5 and  >  8 cmH_2_O, respectively) [10, 11] was assessed by calculating the area under the Receiver Operating Characteristic (ROC) curve, sensitivity, specificity, positive and negative predictive value. To calculate positive and negative predictive values, the prevalence of the condition in the population was assumed to be equal to the prevalence in the sample. Two-tailed P-values < 0.05 were considered for statistical significance.

## Results

### Patient characteristics

Fourteen consecutive patients were enrolled. Patient characteristics at baseline and the duration of the disease are reported in Table 1. Patients were studied after an average total ICU stay of 26 [22; 29] days. At the time of enrolment, patients had been assisted in pressure support ventilation for 6 [3; 9] days. Mechanical ventilation settings and gas exchange on the study day were as follows: PEEP 6 [5; 7] cmH_2_O, FiO_2_ 0.40 ± 0.05; PaO_2_ 103.4 ± 24.7 mmHg, pH 7.45 ± 0.06, PaCO_2_ 41.6 ± 6.8 mmHg, Base excess 4.8 ± 3.9. The level of pressure support, as clinically set by the intensivists caring for the patients, was 10 [8; 10]. The average score on the Richmond agitation-sedation scale was 0 [0; 0]. Diaphragm ultrasound examinations could be performed in all patients, and no patients developed signs of distress during the study.

**Table 1:** Patient characteristics and outcomes at baseline duration of the disease and mechanical ventilation settings on the study day

Variable	Study population (n = 14)
Age (years)	64 ± 7
Male sex—n (%)	13 (93%)
Actual body weight (kg)	92 ± 13
Ideal body weight (kg)	71 ± 8
Body height (m)	1.77 ± 0.09
BMI (Kg/m^2^)	29.3 ± 4.0
SAPS II (points)	37 ± 5
Admission from	
Medical ward	8 (57%)
Emergency department	5 (36%)
Other ICU	1 (7%)
Onset of symptoms to hospital admission (days)	6 [4; 9]
Hospital admission to ICU admission (days)	2 [1; 3]
Non-invasive ventilation before ICU admission (n—%)	8 (57%)
Duration of controlled mechanical ventilation (days)	18 [15; 20]
Duration of PSV before the study day (days)	6 [3; 9]
Length of ICU stay (days)	36 ± 5
ICU mortality	1 (7%)

*N* sample size, *BMI* body mass index, *SAPS II* simplified acute physiology score, 2nd version, *ICU* intensive care unit, *FiO*_*2*_ fraction of inspired oxygen, *BE* base excess, *RASS* Richmond agitation-sedation scale

### Effects of pressure support ventilation changes

Table 2 reports the haemodynamic parameters and indices of respiratory drive and effort during the three steps. No significant changes in the haemodynamic parameters was detected with the reduction in the support level. Reduction of pressure support was associated with a significant increase of respiratory rate, as well as a significant decrease in tidal volume, so that minute ventilation was not modified.

**Table 2:** Haemodynamic parameters and indices of respiratory drive and effort in the different study steps

Variable	Pressure support level			p
	PS 0	PS 5	PS 10	
Mean arterial blood pressure (mmHg)	90 ± 11	88 ± 11	89 ± 14	0.9364
Heart rate (bpm)	93 ± 13	92 ± 12	87 ± 12	0.4143
End-expiratory transmural central venous pressure (mmHg)	5 [− 1; 9]	4 [− 1; 9]	4 [0; 8]	0.4056
Respiratory rate (bpm)	25 ± 6	23 ± 5	19 ± 4°*	0.0069
Tidal volume (mL)	461 ± 58	495 ± 43	560 ± 104°*	0.0032
Minute ventilation (L/min)	11.6 ± 3.3	11.5 ± 2.7	10.7 ± 2.8	0.6569
End-expiratory esophageal pressure (cmH_2_O)	12 [7; 13]	10 [7; 12]	8 [6; 12]	0.1014
End-inspiratory esophageal pressure (cmH_2_O)	0 [− 3; 3]	3 [− 2; 4]*	4 [1; 7]°*	< 0.0001
End-expiratory central venous pressure (cmH_2_O)	14 [12; 19]	14 [10; 16]	12 [9; 15]	0.2938
End-inspiratory central venous pressure (cmH_2_O)	4 [− 1; 11]	5 [1; 12]	8 [3; 14]°*	0.0001
End-expiratory diaphragm thickness (cm)	0.22 ± 0.03	0.22 ± 0.03	0.22 ± 0.03	> 0.9999
End-inspiratory diaphragm thickness (cm)	0.28 ± 0.03	0.26 ± 0.03	0.23 ± 0.03*	0.0079
P0.1 (cmH_2_O)	3.0 ± 1.5	2.0 ± 1.4	1.6 ± 1.2*	0.0418
Pmusc, index (cmH_2_O)	9.6 ± 5.2	5.0 ± 3.6*	2.8 ± 2.4*	0.0026

Data are expressed as mean ± standard deviation or median [interquartile range]

*PS* pressure support

The analysis on the variables recorded over the three different steps (PS 0, PS 5 and PS 10) was performed on all the patients by analysis of variance for repeated measurements, with step as a within-subject factor in case of normally-distributed variables, and the significance of the within-subject factors was corrected with the Greenhouse–Geisser method. Non-parametric variables were analyzed using Friedman test. Pairwise post-hoc multiple comparisons were carried out when appropriate. *p < 0.01 vs. PS 0; °p < 0.01 vs. PS 5

### Respiratory drive and effort

**Fig. 2:**
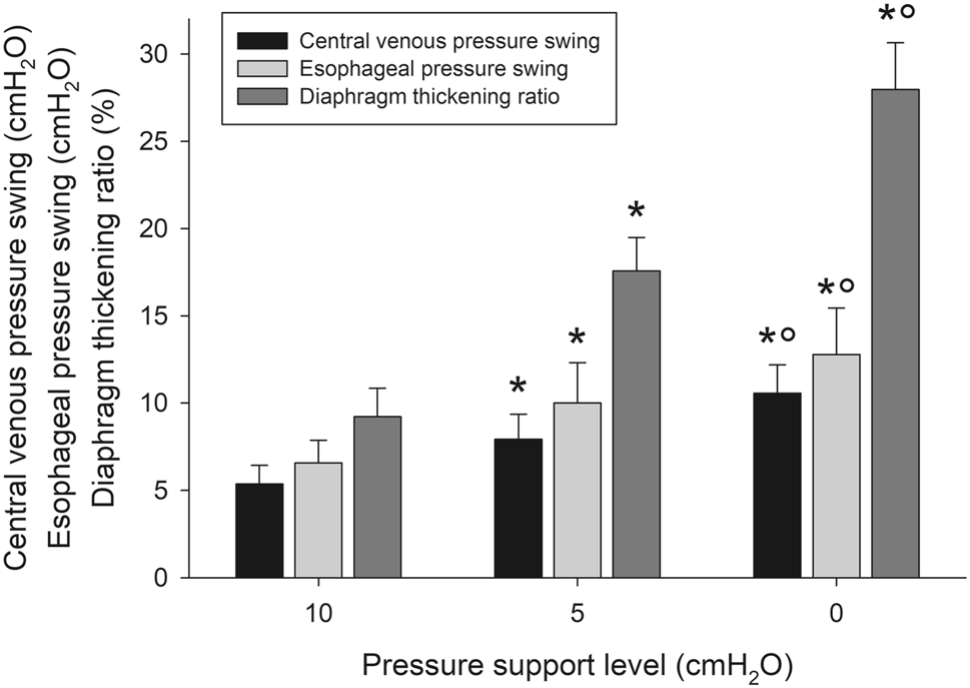
Central venous pressure swing, esophageal pressure swing and diaphragm thickening ratio during the three steps of the study. The analysis on the variables recorded over the three different steps (PS 0, PS 5 and PS 10) was performed on all the patients by analysis of variance for repeated measurements, with step as a within-subject factor in case of normally-distributed variables, and the significance of the within-subject factors was corrected with the Greenhouse–Geisser method. Non-parametric variables were analyzed using Friedman test. Pairwise post-hoc multiple comparisons were carried out when appropriate. *p < 0.01 vs. PS 0; °p < 0.01 vs. PS 5

The progressive reduction of support was not associated with any change in end-expiratory Pes, while end-inspiratory Pes was significantly reduced. As a result, we found a significant increase in ΔPes (5 [3; 8] vs. 8 [4; 13] vs. 12 [6; 16] cmH_2_O, p < 0.0001) (Fig. 2). Similarly, P0.1 and Pmusc,index significantly increased with lowering pressure support.

**Fig. 3:**
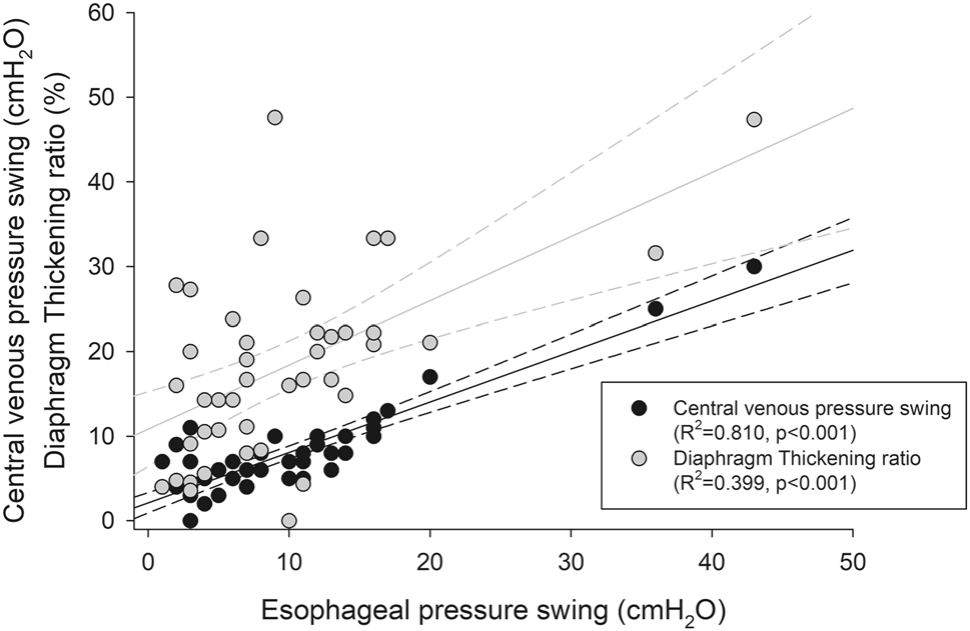
Correlation of central venous pressure swing and diaphragm thickening ratio with the esophageal tidal pressure swing during the different phases of the study. Black dots represent the central venous pressure swing, while gray dots depict the diaphragm thickening ratio. The solid lines represent the linear predictions, while the dashed lines are their 95% confidence interval. The analysis was conducted on all patients by a linear, fixed-effects model for repeated measures to deal with the longitudinal structure of our data set (patients with repeated measurements over time). The extent of the association between variables was expressed as the coefficient of determination (R^2^)

End-expiratory CVP was not statistically different during the different steps of the study, while the reduction of pressure support was associated with a significant decrease in the end-inspiratory CVP. As a result, the decrease in pressure support was associated with a significant increase in the ΔCVP (4 [3; 7] vs. 8 [5; 9] vs. 10 [7; 11] cmH_2_O, p < 0.0001) (Fig. 2). ΔCVP was significantly associated with ΔPes (R^2^ = 0.810, p < 0.001) (Fig. 3).

### Ultrasonographic indices of respiratory effort

The expiratory thickness of the diaphragm was unchanged in the different steps, whereas the inspiratory thickness, and hence the thickening ratio, significantly increased with lowering levels of support (9.2 ± 6.1 vs. 17.6 ± 7.2 vs. 28.0 ± 10.0%, p < 0.0001) (Fig. 2). Diaphragm thickening ratio significantly correlated with ΔPes (R^2^ = 0.399, p < 0.001) (Fig. 3).

In a multivariable, linear, fixed-effects regression, only ΔCVP was significantly associated with ΔPes (coefficient: 1.45 ± 0.16, p < 0.001), while diaphragm TR was not (coefficient: − 0.07 ± 0.05, p = 0.184).

## *Effects of CVP on the* ΔCVP-ΔPes *relationship*

**Fig. 4:**
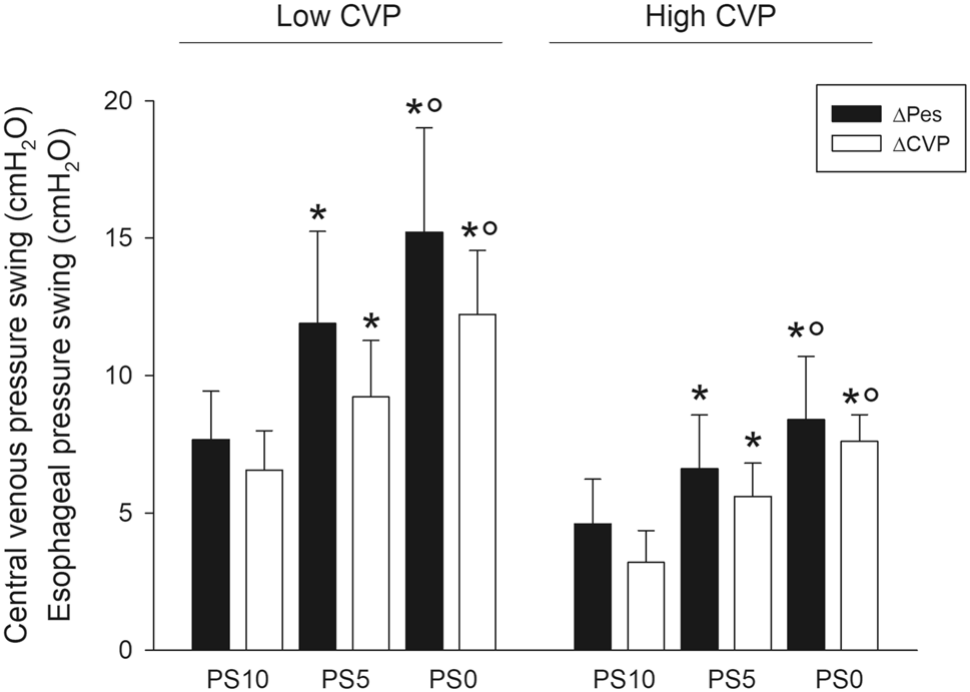
Esophageal and central venous pressure swing in patients with a high or a low central venous pressure during the three steps of the study. The analysis on the variables recorded over the three different steps (PS 0, PS 5 and PS 15) was performed on all the patients by analysis of variance for repeated measurements, with step as a within-subject factor in case of normally-distributed variables, and the significance of the within-subject factors was corrected with the Greenhouse–Geisser method. Non-parametric variables were analyzed using Friedman test. Pairwise post-hoc multiple comparisons were carried out when appropriate. *p < 0.01 vs. PS 0; °p < 0.01 vs. PS 5

The median value of end-expiratory CVP was 14 cmH_2_O; this value was used to dichotomize patients into those with high or low CVP. Figure 4 shows the esophageal and central venous pressure swings in patients with a high or a low central venous pressure during the three steps of the study. The association between ΔCVP and ΔPes was similar in patients with high or low CVP (R^2^ = 0.862, p < 0.001 and R^2^ = 0.817, p < 0.001, respectively). Similar results were found when investigating the effect of transmural CVP (Supplementary Results and Supplementary Figure S1).

### Diagnostic performance of ΔCVP and diaphragm TR for detecting a low or a high inspiratory effort

The areas under the ROC curves for the detection of a low inspiratory effort were 0.783 [0.613; 0.954] for the central venous pressure swing and 0.736 [0.554; 0.918] for diaphragm TR, p = 0.6218 (Supplementary Figure S2). The best cutoffs were 5 cmH_2_O for ΔCVP and 8% for diaphragm TR. As for the detection of a high inspiratory effort, the areas under the ROC curves were 0.743 [0.582; 0.903] for diaphragm TR and 0.866 [0.766; 0.986] for ΔCVP, p = 0.0484. The best cutoffs were 9 cmH_2_O for ΔCVP and 20% for diaphragm TR. Supplementary Table S1 shows the diagnostic performance of the best cutoffs for central venous pressure swing and diaphragm thickening ratio for detecting either a low or a high inspiratory effort.

## Discussion

The main findings of this study are: (1) both the bedside-available ΔCVP and the diaphragm TR were related to the level of patient inspiratory effort, as assessed by the ΔPes; (2) the ultrasonographic assessment of diaphragm thickening yielded an only acceptable estimate of respiratory effort, while the ΔCVP had a much stronger correlation with ΔPes over a broad range of inspiratory efforts; (3) the association between ΔCVP and ΔPes was independent of the values of CVP; (4) ΔCVP has a high specificity and an acceptable sensitivity to detect a high or a low inspiratory effort, as defined by specific threshold of esophageal pressure.

### Mechanical ventilation in COVID-19 patients

Several recommendations have been published to guide the ventilator management of patients admitted to the ICU for COVID-19-related acute respiratory failure [2–5]. However, the weaning process has not been fully covered, and suggestions have been made to follow the general criteria for weaning in any type of respiratory failure.

In the Lombardy region, at the peak of the emergency, more than twice the number of preexisting ICU beds were occupied by patients with severe COVID-19 [33]. Contributing to this resource scarcity is the prolonged ventilator dependence and the difficult weaning associated to prolonged prone positioning, deep sedation and use of muscle relaxants and corticosteroids [1]. Given the lack of ICU beds, many clinicians attempted weaning from mechanical ventilation, well aware of the difficult balance between the detrimental and beneficial effects of spontaneous breathing effort [12], i.e. the potential protection against diaphragm contractile dysfunction [34, 35] and the possibility of self-inflicted lung injury from an excessive respiratory drive [36].

However, neither a breathing pattern with utilization of accessory muscles, nor a rapid or shallow breathing, or the inspection of ventilator vaweforms allow any quantitative assessment of breathing effort [13, 37] and an appropriate bedside monitoring of inspiratory effort is strongly required.

### Bedside estimation of patient inspiratory effort

When the inspiratory muscles contract, the size of the ribcage increases, reducing pleural pressure, which can be accurately estimated at the bedside by the assessment of ΔPes [38, 39]. We recently demonstrated how, in patients undergoing assisted mechanical ventilation after acute hypoxemic respiratory failure, this parameter is strongly correlated with gold-standard indices of respiratory effort over a relatively wide range of loading conditions [40].

### Inspiratory effort and diaphragm ultrasound

Diaphragm ultrasonography has been recently suggested to monitor respiratory workload [22, 23], as the inspiratory thickening ratio has shown fair correlation to respiratory pressure generation (i.e. the ΔPes) [40–43]. The non-invasive nature, low-costs, steep learning curve and straightforward calculations are its main advantages.

As expected, we found that TR increased with decreasing levels of support. However, the correlation despite being statistically significant, was far from ideal. First of all, TR is insensitive to duration and frequency of contractions. Moreover, TR may differ from inspiratory effort in two directions: a low TR in the presence of a high ΔPes, and the opposite. The first case (high inspiratory effort, low diaphragm thickening) can arise when diaphragm dysfunction is present [40]. Less intuitive is the second condition, i.e. the coexistence of a high diaphragm thickening with a low inspiratory effort. The relationship between diaphragm thickening and pressure generation may in fact depend on the pattern of thoracoabdominal motion, so that more thickening is expected for any given ΔPes when the inspiration is predominantly accommodated by descent of the diaphragm rather than expansion of the ribcage. Although the thoracoabdominal pattern was not recorded in the present investigation, we can speculate that this issue might be a factor contributing to our results.

### Inspiratory effort and central venous pressure swing

Since the superior vena cava is a highly compliant, intrathoracic vein, indwelling central venous catheters have been used to estimate the respiratory effort without resorting to additional catheters. That CVP and Pes fluctuate during ventilation in a similar way is not a new finding [44]. During positive-pressure breathing in passive conditions, tidal changes of Pes and CVP were almost identical in size, and linearly correlated [19].

Quite surprisingly, however, the use of ΔCVP to estimate inspiratory effort has seldom been reported, and is generally not used in the everyday clinical practice. Flemale et al. measured CVP and Pes with identical fluid-filled systems in healthy volunteers during inspiratory efforts against a closed airway in seated, supine, right lateral and left lateral positions, and concluded that CVP reflects Pes, with a ΔCVP/ΔPes ratio close to the unity [18]. Similar results were recently found in 10 patients undergoing progressive reduction of inspiratory pressure support, in whom the ratio of ΔCVP to ΔPes was on average 1.1, with a mean difference of 1 cmH_2_O [20]. We found that ΔCVP was an adequate estimate of inspiratory muscle force generation across a wide range of loading conditions.

Potential factors which may affect the concordance between Pes and CVP include the fact that CVP is measured with a fluid-filled system, while Pes is measured with an air-filled system. While fluid-filled systems have a theoretical better frequency response, only small differences were found between the two systems [45]. Moreover, a poor transmission of the pleural pressure changes into the superior vena cava is also possible, and a reduction of the compliance of the vena cava has been suggested as a mechanism [18]. Since the filling of the superior vena cava may potentially influence its mechanical characteristics [21], a secondary outcome of the present investigation was to investigate the effect of CVP (both as the absolute and the transmural value) on the correspondence between ΔCVP and ΔPes. We found that the association between the two variables was significant independently of the value of CVP.

Indeed, it is known that weaning-induced pulmonary oedema is characterized by a progressive increase in transvascular pressure, which is then the cause of the clinical picture [46–48]. In our population, the three-step, sequential reduction of the pressure support levels was not associated with any statistically significant increase in the transmural CVP, likely because none of the patients had severe heart disease [48]; moreover, even at the lowest level of support (PS0), a CPAP was always guaranteed [49].

### Diagnostic performance

We investigated the diagnostic characteristics of central venous pressure swing and diaphragm thickening ratio in detecting a low or a high inspiratory effort, as defined by widely accepted threshold values of esophageal pressure swing [10, 11]. Concerning a low level of inspiratory effort, both indices had a similar diagnostic performance, as assessed by the area under the ROC curve. As far as the detection of a high inspiratory effort is concerned, ΔCVP had a significantly higher overall diagnostic performance as compared to diaphragm TR. Central venous pressure swing proved a specific rather than a sensitive test, both for low and high inspiratory effort: this means that it can effectively be used to rule in the specific condition with virtually no false negatives and an overall acceptable discriminative ability (i.e. a CVP swing < 5 cmH_2_O rules in a low inspiratory effort, whereas a CVP swing > 8 cmH_2_O rules in a high inspiratory effort).

### Clinical impact

Monitoring of respiratory drive and effort is increasingly recognized as necessary during assisted mechanical ventilation, as both excessively low and high levels can have detrimental consequences in terms of lung injury and diaphragm myotrauma [11]. However, despite the availability of monitoring techniques (mainly used for research purposes), inspiratory effort is seldom measured directly [50]. In the current investigation we showed how the inspiratory swing of CVP, a near-ubiquitous monitoring in the ICU, can be used to track inspiratory effort with an acceptable accuracy. Since monitoring of inspiratory effort was suggested for patients at higher risk of complications from injurious breathing [10], such as those with more severe lung injury or systemic inflammation, and patients with COVID-19-associated acute respiratory failure have a form of injury that was found similar to patients with ARDS unrelated to COVID-19 [51], we speculate that using ΔCVP to assess inspiratory effort may be a useful tool in other patients with acute hypoxemic respiratory failure.

### Limitations

Our study has some limitations: first, we studied a relatively small population, which is, however, comparable with that of other physiological studies [43, 52]. Moreover, patients were observed over a limited time-frame: we are unaware if and how much the results would change over time and their clinical relevance. The level of pressure support at the time of enrolment in the study was set by the clinician according to local recommendations. Indeed, it was shown how the clinical setting of pressure support often results in higher levels than needed by the patients to preserve respiratory muscle function while at the same time preventing passive lung inflation, and several physiological model based decision support systems may help in the selection of appropriate ventilator settings [53].

In the current investigation, esophageal pressure was used as an index of global inspiratory effort. Indeed, we did not measure transdiaphragmatic pressure, which is the gold-standard measurement of the pressure-generating ability of the diaphragm. Despite the exclusion of patients with a story of COPD, intrinsic PEEP was not measured, and we cannot exclude the effect of hyperinflation on our findings. While the population included in the present study suffered from COVID-19-related acute respiratory failure and the virtual absence of any relevant comorbidity in the patients warns against direct extrapolation of our results to other groups of patients, the strong physiological basis of our findings make us speculate in favour of a possible utility of these measurements in other categories of patients. Further studies are however warranted to verify this point.

## Conclusions

In conclusion, the present study provides a possible physiologic guidance for the bedside monitoring of inspiratory effort of COVID-19 patients. We found that ΔCVP is an easily available and reliable surrogate of respiratory muscle pressure generation, and that it may perform better than the ultrasonographic assessment of the diaphragm, across a wide range of inspiratory efforts and independently of the patient filling state.

## Supplementary Information

Below is the link to the electronic supplementary material.

Supplementary Information 1 (DOCX 520 kb)

## Data Availability

Data will be made available by the corresponding author upon reasonable request.
