# Supplementary material for: Central venous pressure swing outperforms diaphragm ultrasound as a measure of inspiratory effort during pressure support ventilation in COVID-19 patients
Source: J Clin Monit Comput. 2021 Feb 26;36(2):461–71. doi: 10.1007/s10877-021-00674-4 (PMC7908005; doi:10.1007/s10877-021-00674-4)
Supplement: Supplementary file 1 — Supplementary Information 1 (DOCX 520 kb) [file 10877_2021_674_MOESM1_ESM.docx]

Sergio Lassola^a^, Sara Miori^a^, Andrea Sanna^a^, Alberto Cucino^a^, Sandra Magnoni^a^, Michele Umbrello^b^

**CENTRAL VENOUS PRESSURE SWING OUTPERFORMS DIAPHRAGM ULTRASOUND AS A MEASURE OF INSPIRATORY EFFORT DURING PRESSURE SUPPORT VENTILATION IN COVID-19 PATIENTS**

From the:

a: SC Anestesia e Rianimazione 1, Ospedale Santa Chiara, Trento, Italy

b: SC Anestesia e Rianimazione II, Ospedale San Carlo Borromeo, ASST Santi Paolo e Carlo, Milano, Italy

**Electronic supplementary material**

**SUPPLEMENTARY RESULTS**

*Effects of transmural CVP*

The median value of end-expiratory transmural CVP was 4 cmH_2_O; this value was used to dichotomize patients into those with high or low transmural CVP. Supplementary figure S1 shows the esophageal and central venous pressure swings in patients with a high or a low transmural central venous pressure during the three steps of the study.

The association between ΔCVP and ΔPes was similar in patients with high or low transmural CVP (R^2^=0.795, p<0.001 and R^2^=0.789, p<0.001, respectively). Transmural CVP was not correlated with the value of ΔCVP (R^2^=0.002, p=0.965) or the difference between ΔCVP and ΔPes (R^2^=0.060, p=0.983).

**Supplementary table S1: Diagnostic performance of the best cutoffs for central venous pressure swing and diaphragm thickening ratio for detecting either a low or a high inspiratory effort (arbitrarily defined as an esophageal pressure swing <5 and >8 cmH_2_O, respectively)**

|  | Sensitivity  % [95% CI] | Specificity  % [95% CI] | PPV  % [95% CI] | NPV  % [95% CI] |
| --- | --- | --- | --- | --- |
| *Low inspiratory effort* |  |  |  |  |
| CVP swing <5 cmH_2_O | 58.3 [27.7; 84.8] | 90.0 [73.5; 97.9] | 70.0 [34.8; 93.3] | 84.4 [67.2; 94.7] |
| Diaphragm TR <8% | 41.7 [15.2; 72.3] | 90.0 [73.5; 97.9] | 62.5 [24.5; 91.5] | 79.4 [62.1; 91.3] |
| *High inspiratory effort* |  |  |  |  |
| CVP swing >9 cmH_2_O | 57.9 [33.5; 79.7] | 91.3 [72.0; 98.9] | 84.6 [54.6; 98.1] | 72.4 [52.8; 87.3] |
| Diaphragm TR >20% | 68.4 [43.4; 87.4] | 73.9 [51.6; 89.8] | 68.4 [43.4; 87.4] | 73.9 [51.6; 89.8] |

Data are expressed as the estimate of the diagnostic parameter [95% confidence interval]; CVP: central venous pressure; TR: Thickening ratio; CI: confidence interval; PPV: positive predictive value; NPV: negative predictive value

**Supplementary figure S1 – Esophageal and central venous pressure swing in patients with a high or a low transmural central venous pressure during the three steps of the study.**


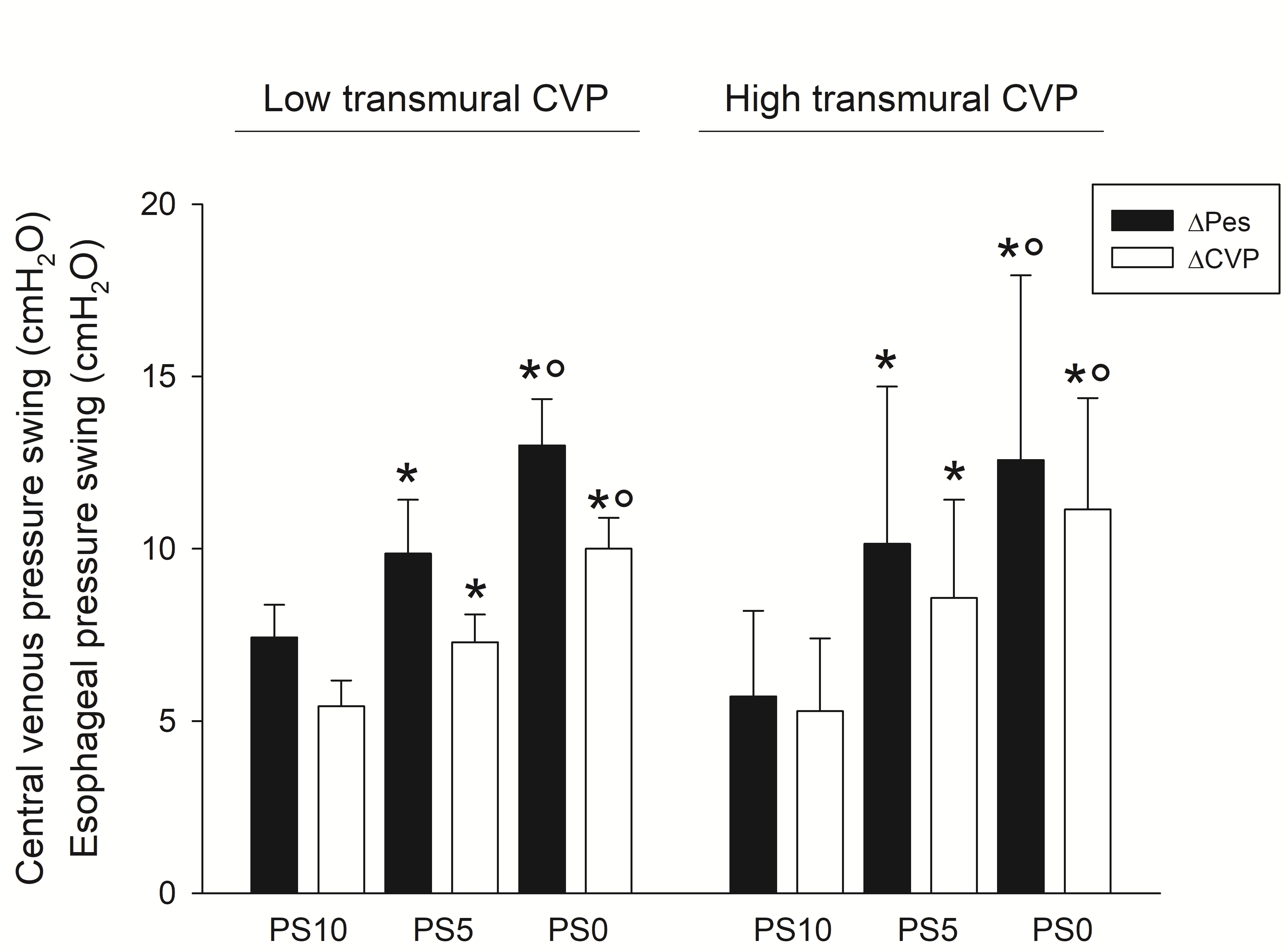


The analysis on the variables recorded over the three different steps (PS 0, PS 5 and PS 15) was performed on all the patients by analysis of variance for repeated measurements, with step as a within-subject factor in case of normally-distributed variables, and the significance of the within-subject factors was corrected with the Greenhouse-Geisser method. Non-parametric variables were analyzed using Friedman test. Pairwise post-hoc multiple comparisons were carried out when appropriate. *p<0.01 vs. PS 0; °p<0.01 vs. PS 5.

**Supplementary figure S2 – Diagnostic performance of the central venous pressure swing and diaphragm thickening ratio for detecting either a low (left panel) or a high (right panel) inspiratory effort (arbitrarily defined as an esophageal pressure swing <5 and >8 cmH_2_O, respectively)**


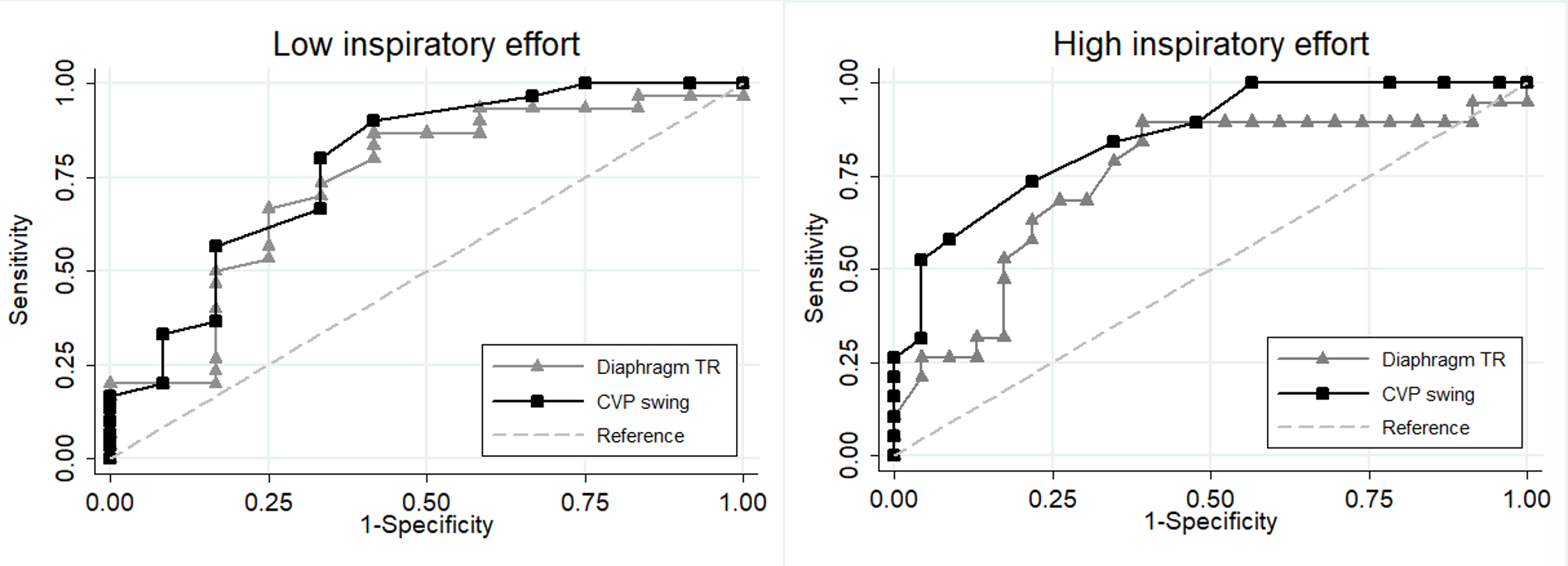


TR: Thickening ratio; CVP: Central venous pressure
